# Role of aberrant metalloproteinase activity in the pro-inflammatory phenotype of bronchial epithelium in COPD

**DOI:** 10.1186/1465-9921-12-110

**Published:** 2011-08-23

**Authors:** Irene H Heijink, Simone M Brandenburg, Jacobien A Noordhoek, Dirk-Jan Slebos, Dirkje S Postma, Antoon J van Oosterhout

**Affiliations:** 1 Laboratory of Allergology & Pulmonary Diseases, Department of Pathology & Medical Biology, University Medical Center Groningen, University of Groningen, The Netherlands; 2 Department of Pulmonology, Groningen Research Institute for Asthma and COPD, University Medical Center Groningen, University of Groningen, The Netherlands

**Keywords:** Cigarette smoke, ADAM17, IL-8, TGF-α, TIMP-2

## Abstract

**Background:**

Cigarette smoke, the major risk factor for COPD, is known to activate matrix metalloproteinases in airway epithelium. We investigated whether metalloproteinases, particularly A Disintegrin and Metalloproteinase (ADAM)17, contribute to increased pro-inflammatory epithelial responses with respect to the release of IL-8 and TGF-α, cytokines implicated in COPD pathogenesis.

**Methods:**

We studied the effects of cigarette smoke extract (CSE) and metalloproteinase inhibitors on TGF-α and IL-8 release in primary bronchial epithelial cells (PBECs) from COPD patients, healthy smokers and non-smokers.

**Results:**

We observed that TGF-α was mainly shed by ADAM17 in PBECs from all groups. Interestingly, IL-8 production occurred independently from ADAM17 and TGF-α shedding, but was significantly inhibited by broad-spectrum metalloproteinase inhibitor TAPI-2. CSE did not induce ADAM17-dependent TGF-α shedding, while it slightly augmented the production of IL-8. This was accompanied by reduced endogenous inhibitor of metalloproteinase (TIMP)-3 levels, suggesting that CSE does not directly but rather indirectly alter activity of ADAM17 through the regulation of its endogenous inhibitor. Furthermore, whereas baseline TGF-α shedding was lower in COPD PBECs, the early release of IL-8 (likely due to its shedding) was higher in PBECs from COPD than healthy smokers. Importantly, this was accompanied by lower TIMP-2 levels in COPD PBECs, while baseline TIMP-3 levels were similar between groups.

**Conclusions:**

Our data indicate that IL-8 secretion is regulated independently from ADAM17 activity and TGF-α shedding and that particularly its early release is differentially regulated in PBECs from COPD and healthy smokers. Since TIMP-2-sensitive metalloproteinases could potentially contribute to IL-8 release, these may be interesting targets to further investigate novel therapeutic strategies in COPD.

## Introduction

Chronic Obstructive Pulmonary Disease (COPD) is characterized by ongoing airway inflammation, which is associated with pulmonary emphysema and/or airway remodeling. This results in airway obstruction and accelerated lung function decline. Although smoking is the major cause of COPD, it is still unclear how different phenotypes of COPD develop from the same exposure to cigarette smoke. When inhaled, cigarette smoke first encounters the airway epithelium that constitutes a barrier to environmental substances. Aberrant repair to smoke-induced injury may lead to remodeling of airway epithelium, an important feature of COPD that includes squamous metaplasia and mucous cell hypertrophy. This may reduce epithelial barrier function in association with increased pro-inflammatory epithelial activity.

Matrix metalloproteinase (MMP)s and A Disintegrin and Metalloproteinase (ADAM)s are thought to play an important role in airway remodeling in various respiratory diseases, including COPD [1]. The disintegrin domain of ADAMs is involved in regulation of integrin-mediated cell adhesion, while the metalloproteinase domain can induce surface cleavage of heparan sulfate proteoglycans (HSPG), growth factors, cytokines, extracellular matrix proteins and intercellular contact proteins [2]. Various ADAMs are expressed in bronchial epithelium of the human lung [3]. Moreover, elevated MMP-2, -9, -12 and -14 levels have been reported in COPD before and/or during exacerbations and in mouse models [4-11]. Therefore, metalloproteinases may be considered as potential drug targets for the treatment of COPD.

Interestingly, cigarette smoke has been shown to reduce the expression of endogenous tissue inhibitors of metalloproteinase (TIMP)s and to activate ADAM17, which results in TGF-α shedding in the airway epithelial cell line NCI-H292 [12-15].

This may have important implications for COPD. TGF-α is a well-known ligand of the EGF receptor (EGFR), which was shown to be involved in ADAM17-dependent mucus hypersecretion and IL-8 production [12-16]. IL-8 is a chemoattractant for neutrophils [17], which play a central role in the pathogenesis of COPD [18]. Indeed, current and ex-smoking COPD patients display higher IL-8 levels in bronchial epithelium than healthy smokers [19,20].

Despite emerging implications for ADAMs and MMPs in COPD, little is known about their regulation, specific actions in airway epithelium and role in COPD pathogenesis. We hypothesized that aberrant metalloproteinase activity, in particular activity of the well-known TGF-α sheddase ADAM17, contributes to increased epithelial pro-inflammatory responses to cigarette smoke in COPD. We studied the expression of specific TIMPs and used pharmacologic inhibitors to study the involvement of ADAMs and MMPs in the release of cytokines that are relevant to COPD, e.g. TGF-α and IL-8. We did so in the presence and absence of cigarette smoke extract (CSE) and compared primary bronchial epithelial cells (PBECs) from COPD patients and epithelium from smoking and non-smoking healthy subjects. Our results demonstrate that IL-8 secretion is regulated independently from ADAM17 activity and TGF-α shedding and that particularly the early release of IL-8 is higher in COPD than healthy smokers. Our data further suggest that reduced TIMP-2 levels may contribute to these differences.

## Methods

### Epithelial cell culture

PBECs were obtained from 8 severe COPD patients with GOLD stages III and IV [21] (inclusion based on ≥10 pack-years of smoking, FEV_1 _<50% of predicted, FEV_1_/FVC<70%, median age 56, range 54-65 years, see table I for patient characteristics) from bronchial brushings by bronchoscopy using a fiberoptic bronchoscope according to standard guidelines [22]. Furthermore, we used bronchial epithelial cell cultures from 9 healthy smoking donors (age 48 (39-60) years) as well as 8 healthy non-smokers (age 54 (43-76) years). These cultures were derived from 7 smoking and 5 non-smoking individuals from Lonza (Walkersville, MD) and from bronchial brushings in 2 smoking and 3 non-smoking individuals. TGF-α (2 hrs) and IL-8 (24 hrs) levels were not different in epithelial cells derived from bronchial brushings when compared to cells derived from Lonza (additional file 1, Figure S1). The Medical Ethics Committee of our center approved the study. Signed informed consent was given by participants. Epithelial cultures of all subjects were established similarly as described previously [23]. In short, cells were grown in 2.5 ml hormonally-supplemented bronchial epithelium growth medium (BEGM, Lonza), containing 100 U/ml penicillin and 100 μg/ml streptomycin, in collagen/fibronectin-coated flasks. Cells were passaged using trypsin and further cultured for approximately 2-3 weeks until use at passage 2. We observed that primary epithelial cells from bronchial brushings grown in BEGM all stain positive for pan-cytokeratin (clone AE1/AE3; DAKO) after 2 passages (data not shown). Cells were seeded into 24-well plates at a density of 1 × 10^5^/well, and grown for 3 additional days. Before experimentation, at 90% confluency, BEGM was replaced basal medium (BEBM, Lonza) containing 0.5% FCS and cells were rendered quiescent overnight. Cell viability was evaluated by Trypan Blue staining. 0.5% FCS did not affect cell viability (data not shown).

### Preparation of cigarette smoke extract

Cigarette smoke extract (CSE) was prepared as described previously [24]. In short, Kentucky 2R4F research-reference cigarettes (The Tobacco Research Institute, University of Kentucky, Lexington, KY) were used without filter. Smoke from 2 cigarettes was bubbled through 25 ml medium (100% CSE). The extract was prepared freshly, sterilized using a 0.22 μm filter and used within 30 min.

### Chemical reagents and stimulation of the cells

Cells were pretreated for 5 min with broad-spectrum metalloproteinase inhibitor TAPI-2 (Calbiochem, Omnilabo International BV, Breda, The Netherlands), the dual specific ADAM17 and ADAM10 inhibitor GW280264 or the specific ADAM10 inhibitor GI254023 (both kindly provided by M. Johnson, GSK, UK) in a concentration of 2.5 μM, based on their effects in a dose-response curve (data not shown), or for 30 min with neutralizing α-EGFR (2 μg/ml, clone 225, Perbio Science B.V., Etten-Leur, The Netherlands), specific EGFR tyrosine kinase inhibitor AG1478 (2 μM, Calbiochem) or vehicle (medium/DMSO). Subsequently, cells were exposed to 0.1, 2.5 or 5% CSE or PMA (5 ng/ml, Sigma-Aldrich, St. Louis, MO, USA) for 2 hrs (early release) or 24 hrs (production) and harvested for RNA isolation or cell lysate preparation. Cell viability was evaluated by Trypan Blue staining. The used concentrations of CSE did not affect cell viability (data not shown).

### Measurement of IL-8, TGF-α and TIMP secretion levels

Protein levels were measured in cell-free supernatants using ELISA kits according to the manufacturer's guidelines (R&D systems Europe Ltd., Abingdon, UK).

### Realtime RT-PCR

RNA was isolated and cDNA synthesized as described previously [25]. IL-8 expression was analyzed by real-time PCR using the Taqman^® ^according to the manufacturer's guidelines (Applied Biosystems, Foster City, CA). Validated probes for IL-8 and the house keeping gene β-actin and the TaqMan Master Mix were purchased from Applied Biosystems.

### Immunodetection by western blotting

Total cell lysates were obtained by resuspension of the cells in 1x sample buffer (containing 2% SDS, 10% glycerol, 2% 2-ME, 60 mM Tris-HCl (pH 6.8) and bromophenol blue) and boiling for 5 min. Expression of ADAM17 was analyzed by Western blotting using anti-ADAM17 (R&D systems) and anti-β-actin (Santa Cruz Biotechnology, Santa Cruz, CA) as loading control as described [26]. Relative protein levels were quantified using the gelscan program QuantityOne.

### Statistical analysis

Data were analyzed using the non-parametric rank-sum Mann Whitney U test for analysis between subject groups and the non-parametric Wilcoxon-signed rank test for paired observations within subject groups.

## Results

We performed studies on TGF-α and IL-8 release in PBECs from healthy non-smokers, smokers and patients with COPD.

### Regulation of TGF-α shedding

**Figure 1:**
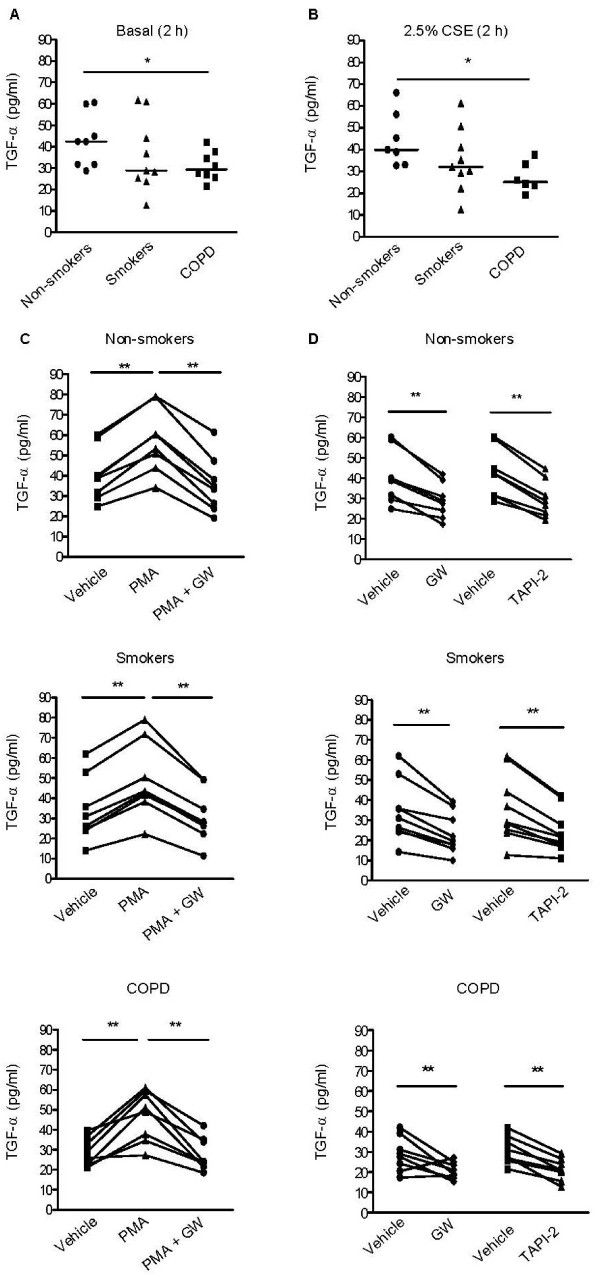
**Basal TGF-α levels are reduced in PBECs from COPD patients versus healthy non-smoking controls, not affected by CSE, induced by PMA and dependent on ADAM17 in PBECs from non-smokers (*n = 8*), healthy smokers (*n = 9*) and COPD patients (*n = 8*)**. Upon overnight growth factor-deprivation, total cell lysates were prepared or cells were pre-treated with and without GW280264 (2.5 μM), TAPI-2 (2.5 μM) or DMSO for 30 min and incubated with medium, CSE (2.5%) or PMA (5 ng/ml) for 2 or 24 hrs (as indicated). **A**) TGF-α levels are reduced in PBECs from COPD patients versus non-smokers under basal conditions. Absolute values and medians are shown. Significance is indicated (* = p < 0.05). **B**) TGF-α levels are not altered by CSE exposure and TGF-α levels are still reduced in PBECs from COPD patients versus non-smokers upon CSE exposure. Absolute values are shown. Significance is indicated (* = p < 0.05). **C**) Increase in TGF-α levels upon PMA exposure to a similar level in PBECs from the 3 different subject groups, which is inhibited by GW280264. Absolute values are shown. Significance is indicated (** = p < 0.01). **D**) GW280264 and TAPI-2 inhibit basal release of TGF-α in the three different subject groups. Absolute values are shown. Significance is indicated (** = p < 0.01).

**Figure 2:**
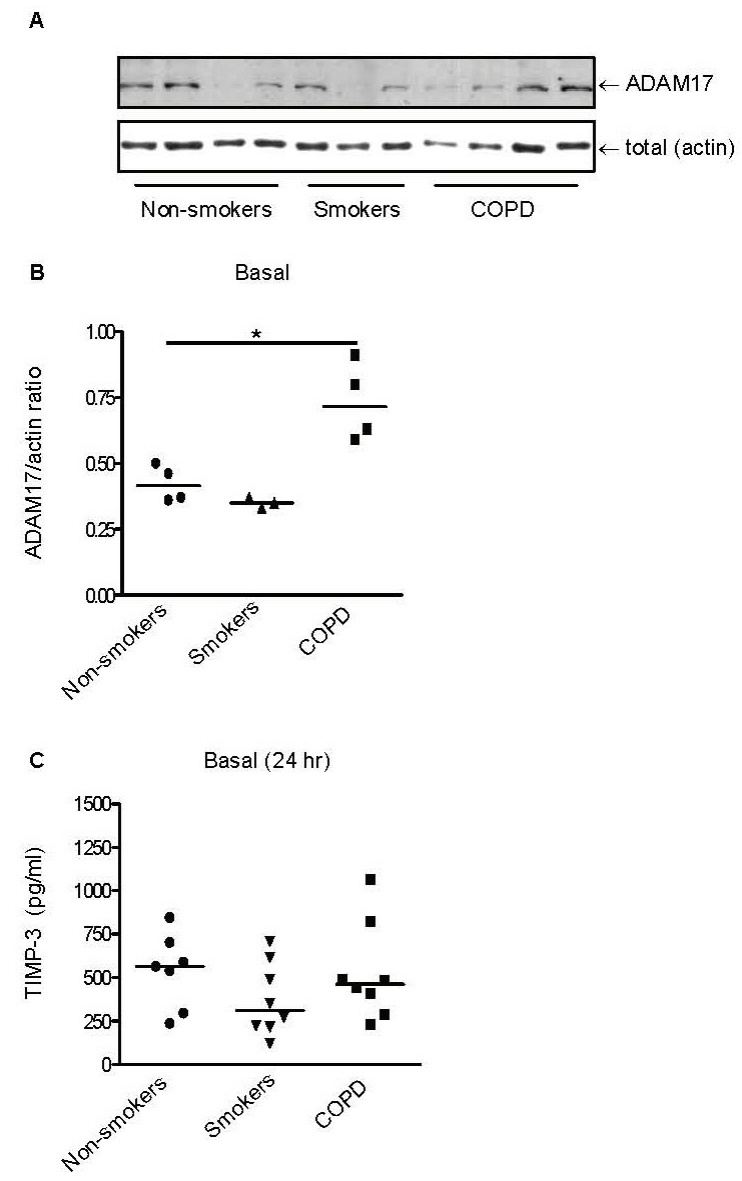
**Differences in baseline TGF-α levels are not related to differences in ADAM17 expression between groups**. PBECs were cultured and seeded in duplicates. Cultures were growth factor-deprived overnight and directly harvested for total cell lysates or incubated for 24 hrs with and without 5% CSE. **A**) ADAM17 and actin were detected by western blotting in PBECs from non-smokers (*n = 4*), healthy smokers (*n = 3*) and COPD patients (*n = 4*) as indicated by arrows. **B**) Densitometry was performed and levels were related to actin levels. The ADAM17/actin ratios and medians are depicted. Significance is indicated (* = p < 0.05). **C**) TIMP-3 levels are not different between groups. Absolute values and medians are shown.

Since we proposed an important role for ADAM17-mediated TGF-α shedding in epithelial IL-8 secretion in COPD, we first studied the regulation of TGF-α shedding by analyzing its early release at 2 hrs. Baseline levels of TGF-α were clearly detectable in culture supernatants of all donor groups. On the other hand, EGFR/ErbB1 ligands EGF and HB-EGF were not detectable at this time point. Unexpectedly, we observed that TGF-α baseline levels were significantly lower in PBECs from COPD patients than from non-smokers (p < 0.05, Figure 1A), while intermediate levels were detected in smokers. In contrast to the data previously described in the cell line NCI-H292 [14], CSE (0.1%, 2.5% and 5%) did not affect TGF-α levels in any of the groups (data shown for 2.5% in Figure 1B), although 5% CSE significantly impacted on trans-epithelial resistance (data not shown). We used the non-specific metalloproteinase activator phorbol myristate acetate (PMA) as a positive control. Unlike CSE, PMA increased TGF-α levels in all groups to a similar extent (~1.5 fold, Figure 1C). The PMA-induced TGF-α shedding was completely blocked by ADAM17/10 inhibitor GW280264 (Figure 1C), but was not affected by specific ADAM10 inhibitor GI254023 (additional file 2, Figure S2), indicating that ADAM17 is the main PMA-induced sheddase of TGF-α upon PBEC activation. Baseline TGF-α levels were also significantly reduced by GW280264, with a reduction of 29 ± 3%, 31 ± 2% and 29 ± 2% in non-smokers, healthy smokers and COPD patients respectively (Figure 1D). TGF-α levels were reduced to a similar extent by TAPI-2, i.e. by 31 ± 2%, 30 ± 3% and 32 ± 5% respectively (Figure 1D). These data indicate that ADAM17 is also the main sheddase of TGF-α under resting conditions. Thus, a reduction in ADAM17 expression or activity could be a plausible explanation for the reduced secretion of TGF-α by COPD epithelium. We analyzed differences in ADAM17 expression by western blotting in 3-4 donors per group. Surprisingly, ADAM17 expression was not lower, but even higher in COPD than in the control groups (Figures 2A, B). Thus, we hypothesized that differences in ADAM17 membrane localization and/or ADAM17 activity could contribute to the differences in TGF-α levels. We tested the secretion of TIMP-3, a potent endogenous inhibitor of ADAM17 activity. However, TIMP-3 levels were not significantly different between the groups (Figure 2C). In further contrast to TGF-α, TIMP-3 levels were significantly reduced upon 24 hrs of CSE exposure (additional file 3, Figure S3).

Together, our data indicate that ADAM-17 is important in constitutive TGF-α shedding and after PMA-stimulation, but that alterations in ADAM17 expression itself or its endogenous inhibitor do not likely contribute to the differences in TGF-α shedding at 2 hours. This is in agreement with the finding that TGF-α levels were still significantly reduced in COPD (20 (25-17) pg/ml) compared to the non-smokers (29 (39-17) pg/ml) group in the presence of GW280264 (p < 0.05).

### Regulation of IL-8 production

**Figure 3:**
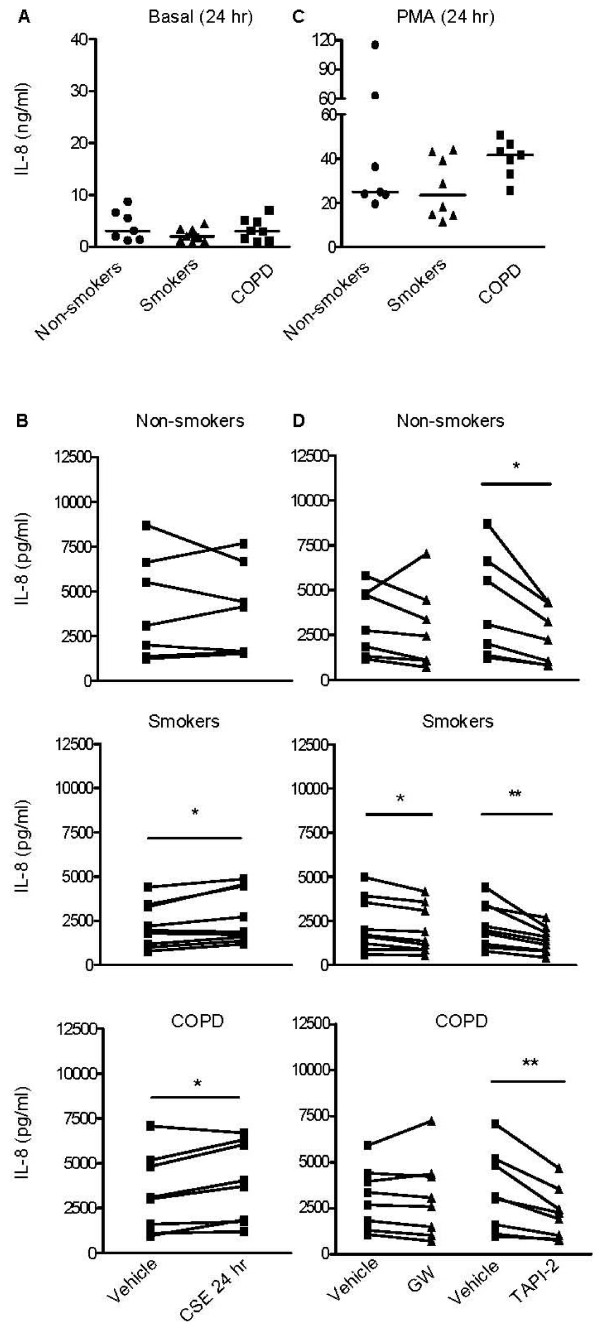
**IL-8 production in PBECs is not dramatically affected by CSE, strongly enhanced by PMA and sensitive to broad-spectrum metalloprotease inhibitor TAPI-2**. PBEC were obtained from non-smokers (*n = 7*), healthy smokers (*n = 9*) and COPD patients (*n = 8*). Cells were growth factor-deprived overnight, pre-treated with and without GW280264 (2.5 μM), TAPI-2 (2.5 μM) or medium/DMSO for 30 min and incubated with and without CSE (2.5%) or PMA (5 ng/ml) for 24 hours. **A**) IL-8 production is not significantly different in the 3 different subject groups under basal conditions. Absolute values and medians are shown. **B**) CSE induces a slight but significant increase in IL-8 production in the healthy smoker and COPD group. Absolute values are shown and significance is indicated (* = p < 0.05). **C**) PMA induces a similar increase in IL-8 production in PBECs from the 3 different subject groups. Absolute values and medians are shown. **D**) TAPI-2 reduced IL-8 production in PBEC from the 3 different subject groups. Significance is indicated. Absolute values are shown. Significance is indicated (* = p < 0.05 and ** = p < 0.01).

Next, we studied the regulation of IL-8 production. As described previously [27], a substantial amount of IL-8 was secreted under baseline conditions (0.8-8.7 ng/ml). In contrast to TGF-α, no significant differences were observed between cells from non-smokers, healthy smokers and COPD patients (Figure 3A). CSE (2.5%) induced a small, but significant increase in IL-8 secretion in COPD patients and healthy smokers, but not in non-smokers (Figure 3B). The use of 5% CSE did not further increase IL-8 levels (additional file 4, Figure S4), whereas PMA induced a strong increase in IL-8 production in all groups (Figure 3C). PMA-induced IL-8 levels tended to be higher in COPD patients than in healthy smokers (p = 0.07, Figure 3C).

In contrast to a previous report in NCI-H292 cells [28], GW280264 did not significantly inhibit basal IL-8 production in PBEC from all donor groups (Figure 3D), either with or without CSE or PMA (data not shown). However, the broad-spectrum metalloproteinase inhibitor TAPI-2 exerted a substantial and significant inhibitory effect in all groups both under basal conditions (39 ± 7%, 34 ± 11% and 33 ± 9% in non-smokers, healthy smokers and COPD respectively, Figure 3D) and upon CSE exposure (additional file 5, Figure S5). Together, these data suggest that a TAPI-2-sensitive metalloproteinase different from ADAM17 (or ADAM10) is involved in baseline and smoke-induced IL-8 production. This is further supported by the observation that the reduction in TGF-α levels was not accompanied by reduced EGFR and phospho-EGFR (i.e. activated EGFR) expression in COPD epithelium, as indicated by western blotting (additional file 6, Figure S6). Thus, shedding of another EGFR/ErbB1 ligand could be involved in the activation of EGFR-downstream signaling and subsequent IL-8 production. Although the well-known EGFR ligand EGF can be shed by ADAM10 [29], GI254023 did not affect IL-8 production by PBEC under any of the conditions (data not shown).

### Regulation of early IL-8 release

In addition to (EGFR-mediated) *de novo *synthesis, we speculated that IL-8 levels may be regulated by a shedding process. Shedding of surface-immobilized IL-8 has been reported in complex with syndecan-1 [30], a proteoglycan that is known to form a complex with various chemokines and that can be cleaved by metalloproteinases [31]. Therefore, we investigated if metalloproteinases contribute to early release of IL-8.

**Figure 4:**
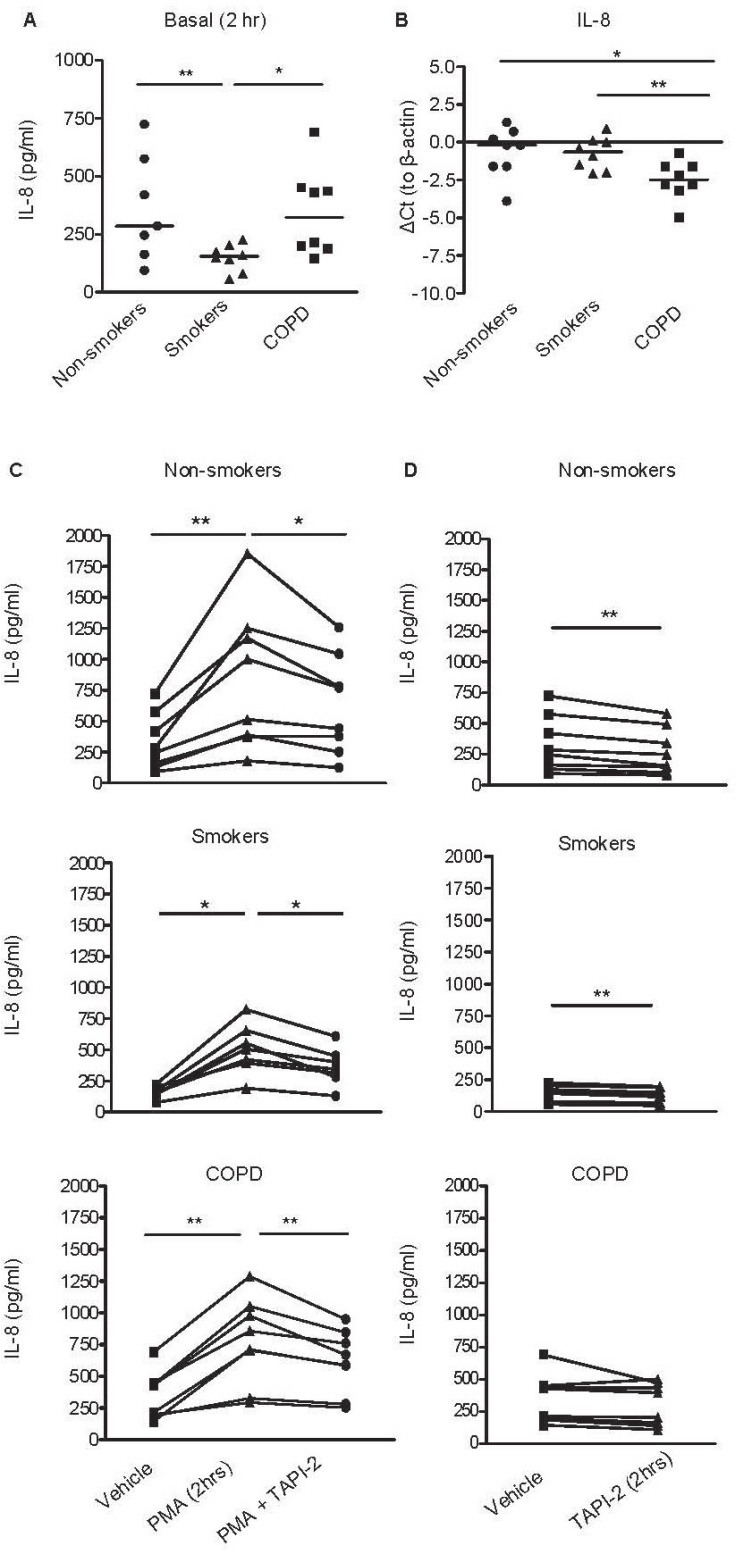
**Early levels of IL-8 are reduced in PBECs from healthy smokers versus COPD patients, induced by PMA and slightly sensitive to TAPI-2**. Cells were growth factor-deprived overnight, pre-treated with medium TAPI-2 (2.5 μM) for 30 min and incubated with medium, CSE (2.5%) or PMA (5 ng/ml) for 2 hrs. **A**) IL-8 levels are significantly reduced in PBECs from smokers versus non-smokers and COPD patients under basal conditions. Absolute values and medians are shown. Significance is indicated (* = p < 0.05 and ** = p < 0.01). **B**) IL-8 mRNA expression was determined by real-time PCR. IL-8 Ct values were subtracted from β-actin Ct values, thus lower ΔCt values reflect lower mRNA levels. ΔCt levels and medians are shown. Significance is indicated (* = p < 0.05 and ** = p < 0.01). **C**) PMA increases IL-8 release in PBEC from the 3 different subject groups, which can be inhibited by TAPI-2. Absolute values are shown and significance is indicated (* = p < 0.05 and ** = p < 0.01). **D**) TAPI-2 significantly inhibits basal IL-8 levels in PBECs from non-smokers and healthy smokers, but not from COPD patients. Absolute values are shown and significance is indicated (** = p < 0.01).

We observed an early basal release of IL-8 (2 hrs), compatible with shedding of syndecan-immobilized IL-8 [31]. Interestingly, this early release was lower in healthy smokers than in COPD (Figure 4A) and non-smokers. Differences in IL-8 mRNA expression (2 hrs) did not correspond to differences in protein IL-8 levels between the groups (Figure 4B), as IL-8 mRNA expression was lower in epithelium from COPD than from healthy smokers and non-smokers. This further supports a role for increased shedding of surface-immobilized IL-8 in COPD.

**Figure 5:**
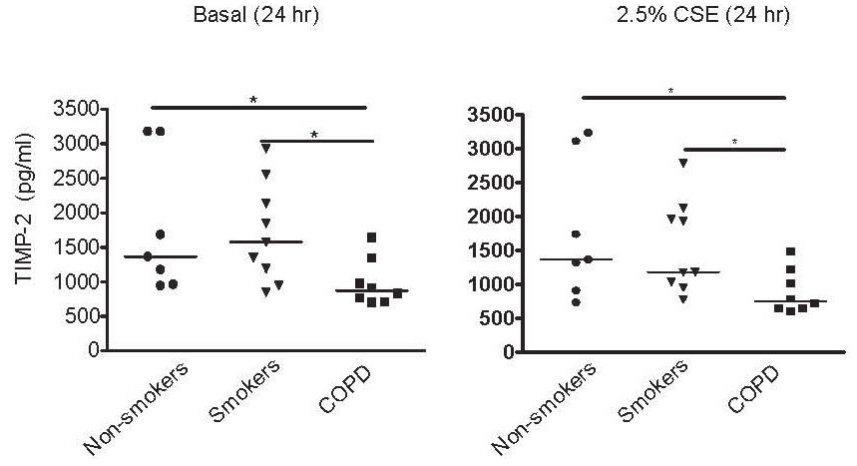
**TIMP-2 levels are reduced in PBEC from COPD patients versus PBECs from the control groups**. Cells were growth factor-deprived overnight and incubated for 24 hrs in **A**) absence and **B**) presence of CSE (2.5%). Absolute values and medians are shown. Significance is indicated (* = p < 0.05).

The early release of IL-8 was not significantly altered by CSE (additional file 7, Figure S7). In contrast, PMA significantly increased the release of IL-8 at 2 hrs (Figure 4C), which was substantially inhibited by TAPI-2, but not by GW280264 or GI254023 (data not shown) in all groups, indicating involvement of metalloproteinases. In line with this, early IL-8 release was inhibited by TAPI-2 (by 19 ± 8%, 12 ± 5% and 12 ± 13% in non-smokers, healthy smokers and COPD patients, respectively, Figure 4D), although this effect was not significant for COPD (p = 0.14). Interestingly, it has been described that TIMP-2 inhibits the actions of MMP-14 (MT1-MMP) [32], a known sheddase of syndecans. We therefore studied TIMP-2 and did the important observation that TIMP-2 levels were significantly reduced in the COPD group versus healthy donors in absence and presence of CSE (Figures 5A and 5B). Thus, loss of this type of regulation of metalloproteinase activity in COPD patients may very well contribute to the increased shedding of immobilized IL-8 in COPD versus healthy smoker epithelium.

## Discussion

MMPs and ADAMs may play a pivotal role in airway remodeling and in pro-inflammatory responses of airway epithelium, as occurs in COPD. We assessed the involvement of metalloproteinases in the secretion of IL-8 and TGF-α, cytokines of importance to COPD development and progression. We investigated the effects of CSE on these cytokines and the possible differences in their regulation in bronchial epithelium from COPD patients, healthy smokers and non-smokers. In striking contrast to data previously published in a cell line [14], our data in primary epithelium show that CSE does not induce ADAM17-dependent TGF-α shedding and only slightly increases IL-8 production. Interestingly, we observed an early IL-8 release by PBEC, likely due to shedding. This early release was higher in PBECs from COPD patients than from healthy smokers and was accompanied by reduced levels of endogenous metalloproteinase inhibitor TIMP-2. Whereas ADAM17 was the main sheddase of TGF-α, we show for the first time that a TAPI-2-sensitive metalloproteinase different from ADAM17 is involved in the regulation of IL-8 release, independently from TGF-α shedding.

We demonstrate that TGF-α shedding is strongly dependent on ADAM17 activity, in contrast to IL-8 secretion. Given the implications for TGF-α in epithelial mucus secretion [14], ADAM17 could serve as potential target for new treatment strategies to reduce mucus hypersecretion in COPD. On the other hand, however, ADAM17 may not be a promising target for the attenuation of IL-8-induced neutrophil recruitment.

In contrast to previous observations in NCI-H292 cells [28], IL-8 secretion was not significantly inhibited by ADAM17 inhibition. This stresses the fact that studies in airway epithelial cell lines cannot simply be extrapolated to primary cells and should be interpreted carefully. More importantly, our data suggest that another, yet unidentified TAPI-2-sensitive metalloproteinase is involved in IL-8 production by airway epithelium. Differences in the regulation of this specific metalloproteinase may contribute to the aberrant pro-inflammatory responses of COPD epithelium. Therefore, it is of interest to identify this metalloproteinase in future studies, offering potential leads for novel therapeutic strategies in COPD.

TGF-α and IL-8 levels were also differentially regulated by CSE, the release of TGF-α being unaffected, while IL-8 production (24 hrs) was slightly increased in COPD patients and healthy smokers. Previously, Kode *et al *have shown a stronger CSE-induced increase of IL-8 in primary epithelium [33]. The dissimilarity between the studies may be attributed to differences in smoke extract preparation and to the fact that Kode *et al *used small airway epithelial cells from only a single healthy non-smoker. We observed that the modest upregulatory effect of CSE on IL-8 levels was accompanied by a significant reduction in TIMP-3 levels upon CSE exposure. Besides ADAM17, TIMP-3 can affect several sheddases, including MMP-9, MMP-14 (MT1-MMP), well-known sheddases of EGFR ligands [34,35]. We speculate that these TIMP-3-sensitive sheddases may thus be involved in the upregulatory effect of CSE on IL-8 production.

With respect to differential epithelial responses in non-smokers, healthy smokers and COPD patients, shedding of TGF-α (both baseline and in presence of CSE) was lower in PBEC from COPD patients than from healthy non-smokers, with intermediate levels in healthy smokers. Our data do not support a role for a reduction in functional ADAM17 in the decreased TGF-α shedding in COPD patients. Thus, reduced *de novo *synthesis, trafficking or surface expression of TGF-α are presumably involved. Since TGF-α has been implicated in epithelial repair, the increased expression of ADAM17 observed on COPD epithelium may be an attempt to increase TGF-α release and facilitate regeneration in a situation of chronic damage.

We found similar baseline and CSE-induced IL-8 production (24 hrs) in all PBEC groups. In contrast to our findings, Schneider *et al *demonstrated increased baseline epithelial IL-8 production in COPD patients [36]. The discrepancy between the studies may be due the different culture procedures, as Schneider *et al *cultured their cells at air-liquid interface. It will be thus of interest to study epithelial IL-8 production upon submerged as well as air-liquid interface culture and to expand our subject groups to COPD patients and control groups matched for current and ex smoking in a future study. Although inflammatory responses in COPD are thought to persist for years after smoking cessation, a limitation in our current study is the fact that only ex-smoking COPD patients were included. Nevertheless, Schulz *et al *[37] were also not able to detect differences in baseline IL-8 production in submerged cultured PBEC from currently smoking COPD patients and smoking controls.

Intriguingly, our data suggest that instead of its production, the shedding of immobilized IL-8 be may be altered in COPD patients versus healthy smokers. The early release of IL-8 was significantly higher in COPD than healthy smokers, and IL-8 mRNA expression did not correspond to this differential IL-8 release. On the contrary, differences in IL-8 mRNA expression did relate to differences in TGF-α levels between groups. Thus, it is possible that the early release of IL-8 obscures potential TGF-α-mediated effects on its transcriptional regulation in PBECs. The early IL-8 release was not affected by ADAM17/10 inhibitor GW280264, but slightly inhibited by TAPI-2. Differences between groups were still observed in the presence of TAPI-2, indicating that TAPI-2-insensitive metalloproteinases could also contribute to this early release. Based on our findings and literature data [30], we suggest that this may be due to differential shedding of syndecan-bound IL-8. TAPI-2-insensitive MMP-14 has been identified as sheddase of syndecan-1, and this effect was inhibited by TIMP-2 expression [31,32]. Decreased expression of TIMP-2 mRNA has previously been reported in COPD versus control lung tissue [38] along with increased MMP-14 levels and/or activity in COPD patients [8]. We observed that TIMP-2 levels were significantly lower in PBECs from COPD than from healthy non-smokers and smokers. Thus, lower TIMP-2 levels may be specific for disease and not related to long-term smoking.

Together, our data suggest that reduced TIMP-2 levels in COPD may lead to altered epithelial MMP activity and shedding of syndecan-bound chemokines (e.g. IL-8). It will therefore be of interest to study TIMP-2 levels in sputum or BAL fluid from COPD patients in future studies and test whether these are related to changes in MMP-14 activity, IL-8 levels and neutrophilia. Furthermore, our data suggest that cigarette smoke does not directly alter the activity of ADAMs and MMPs in airway epithelium, but it may indirectly regulate their activity through their endogenous inhibitors, i.e. TIMP-3 (but not TIMP-2).

## Conclusions

Our data indicate that IL-8 secretion is regulated independently from ADAM17 activity and TGF-α shedding and that particularly its early release is differentially regulated in PBECs from COPD and healthy smokers. Our data further suggest that reduced TIMP-2 levels may contribute to the increased shedding of IL-8 in COPD, leading to increased activity of metalloproteinases sensitive to TIMP-2, e.g. MMP-9 and -14. These metalloproteinases may thus be potential targets for novel therapeutic strategies to reduce airway inflammation in COPD.

## Abbreviations

ADAM: A Disintegrin and Metalloproteinase; COPD: Chronic obstructive pulmonary disease; CSE: cigarette smoke extract; EGFR: EGF receptor; HSPG: heparan sulfate proteoglycans; MMP: Matrix metalloproteinase; PBECs: primary bronchial epithelial cells; PMA: phorbol myristate acetate; ROS: reactive oxygen species; TIMP: tissue inhibitor of metalloproteinase.

## Competing interests

IH, SB, JN, DS and AVO declare that they have no competing interests. DP has received reimbursements, fees, funding and/or salary from AstraZeneca, Boehringer Ingelheim, Chiesi, GSK, Nycomed and TEVA. DP does not hold any stocks or shares in an organization that may in any way gain or lose financially from the publication of this manuscript, either now or in the future. DP does not hold or currently apply for any patents relating to the content of the manuscript. DP has not received reimbursements, fees, funding, or salary from an organization that holds or has applied for patents relating to the content of the manuscript. DP does not have any other financial interests.

## Authors' contributions

IH drafted the manuscript and participated in the design, coordination and statistical analysis of the study. SB performed the experiments and statistical analyses. JN helped with cell culture and performance of the experiments. DS was involved in the recruitment of the patients and performed the bronchial brushings. DP was involved in the recruitment of patients, participated in the design of the study and helped to draft the manuscript. AVO participated in the design and coordination of the study and helped to draft the manuscript. All authors read and approved the final manuscript.

## Supplementary Material

Additional file 1 **Baseline TGF-α and IL-8 levels are not significantly different between epithelial cells (healthy smokers and non-smokers) obtained by bronchial brushings (brush) and normal human bronchial epithelium (NHBE) derived from Lonza**. PBEC were growth factor-deprived overnight, pre-treated and incubated with medium. **A**) TGF-α levels at 2 hrs. Absolute values and medians are shown. **B**) IL-8 levels at 24 hrs. Absolute values and medians are shown. Click here for file

Additional file 2 **TGF-α shedding in PBECs from COPD patients**. PBECs were growth factor-deprived overnight and treated with and without GI254023 (2.5 μM) or GW280264 (2.5 μM) for 2 hrs. Absolute TGF-α levels and medians are shown. Click here for file

Additional file 3 **TIMP-3 levels are significantly reduced by CSE exposure in PBECs from smokers and COPD, but not from healthy controls**. PBECs were cultured and seeded in duplicates. Cultures were growth factor-deprived overnight and directly harvested for total cell lysates or incubated for 24 hrs with and without 5% CSE. Absolute values are shown. Significance is indicated (* = p < 0.05 and p**=<0.01). Click here for file

Additional file 4 **IL-8 secretion in PBECs is not significantly different upon exposure to 5% CSE compared to 2.5% CSE**. PBEC from non-smokers (circles), healthy smokers (squares) and COPD patients (triangles) were growth factor-deprived overnight and incubated with medium or CSE (2.5% and 5%) for 24 hrs. Exposure to 5% CSE does not further increase IL-8 levels in PBECs from smokers/COPD patients nor induce an increase in PBECs from non-smokers. Absolute values are shown. Click here for file

Additional file 5 **CSE-induced IL-8 secretion in PBECs is significantly inhibited by TAPI-2**. PBEC from non-smokers, healthy smokers and COPD patients were growth factor-deprived overnight, pre-treated with and without TAPI-2 (2.5 μM) and incubated with medium or 2.5% CSE for 24 hrs. TAPI-2 significantly inhibits CSE-induced IL-8 levels in PBEC. Absolute values are shown. Significance is indicated (* = p < 0.05 and ** = p < 0.01). Click here for file

Additional file 6 **Basal phospho-EGFR levels are not different between the subject groups**. PBECs from non-smokers (*n = 4*), healthy smokers (*n = 4*) and COPD patients (*n = 4*) were growth-factor deprived overnight and total cell lysates were prepared. EGFR and phospho-EGFR detected by western blotting using anti-phospho-EGFR (1173 tyrosine residue) and anti-EGFR (Santa Cruz Biotechnology, Santa Cruz, CA). Densitometry was performed and (phospho-)EGFR levels were related to EGFR. Ratios and medians are depicted. Click here for file

Additional file 7 **Early levels of IL-8 are not induced by CSE in PBECs**. Cells were growth factor-deprived overnight and incubated with medium or 2.5% CSE for 2 hrs. CSE has no effect on IL-8 levels in PBEC from the 3 different subject groups. Absolute values are shown. Click here for file

## References

[B1] Paulissen G Rocks N Gueders MM Crahay C Quesada-Calvo F Bekaert S Hacha J El HM Foidart JM Noel A Cataldo DD Role of ADAM and ADAMTS metalloproteinases in airway diseases Respir Res 2009 10 127 10.1186/1465-9921-10-127 20034386 PMC2805617

[B2] White JM ADAMs: modulators of cell-cell and cell-matrix interactions Curr Opin Cell Biol 2003 15 598 606 10.1016/j.ceb.2003.08.001 14519395

[B3] Dijkstra A Postma DS Noordhoek JA Lodewijk ME Kauffman HF Ten Hacken NH Timens W Expression of ADAMs ("a disintegrin and metalloprotease") in the human lung Virchows Arch 2009 454 441 9 10.1007/s00428-009-0748-4 19255780

[B4] Baraldo S Bazzan E Zanin ME Turato G Garbisa S Maestrelli P Papi A Miniati M Fabbri LM Zuin R Saetta M Matrix metalloproteinase-2 protein in lung periphery is related to COPD progression Chest 2007 132 1733 40 10.1378/chest.06-2819 17646237

[B5] Beeh KM Beier J Kornmann O Buhl R Sputum matrix metalloproteinase-9, tissue inhibitor of metalloprotinease-1, and their molar ratio in patients with chronic obstructive pulmonary disease, idiopathic pulmonary fibrosis and healthy subjects Respir Med 2003 97 634 9 10.1053/rmed.2003.1493 12814147

[B6] Mercer PF Shute JK Bhowmik A Donaldson GC Wedzicha JA Warner JA MMP-9, TIMP-1 and inflammatory cells in sputum from COPD patients during exacerbation Respir Res 2005 6 151 10.1186/1465-9921-6-151 16372907 PMC1351193

[B7] Demedts IK Morel-Montero A Lebecque S Pacheco Y Cataldo D Joos GF Pauwels RA Brusselle GG Elevated MMP-12 protein levels in induced sputum from patients with COPD Thorax 2006 61 196 201 10.1136/thx.2005.042432 16308335 PMC2080750

[B8] Deshmukh HS McLachlan A Atkinson JJ Hardie WD Korfhagen TR Dietsch M Liu Y Di PY Wesselkamper SC Borchers MT Leikauf GD Matrix metalloproteinase-14 mediates a phenotypic shift in the airways to increase mucin production Am J Respir Crit Care Med 2009 180 834 45 10.1164/rccm.200903-0328OC 19661247 PMC2773913

[B9] Valenca SS da HK Castro P Moraes VG Carvalho L Porto LC Emphysema and metalloelastase expression in mouse lung induced by cigarette smoke Toxicol Pathol 2004 32 351 6 10.1080/01926230490431466 15204978

[B10] Seagrave J Barr EB March TH Nikula KJ Effects of cigarette smoke exposure and cessation on inflammatory cells and matrix metalloproteinase activity in mice Exp Lung Res 2004 30 1 15 14967600 10.1080/01902140490252858

[B11] Brajer B Batura-Gabryel H Nowicka A Kuznar-Kaminska B Szczepanik A Concentration of matrix metalloproteinase-9 in serum of patients with chronic obstructive pulmonary disease and a degree of airway obstruction and disease progression J Physiol Pharmacol 2008 59 Suppl 6 145 52 19218638

[B12] Shao MX Nadel JA Dual oxidase 1-dependent MUC5AC mucin expression in cultured human airway epithelial cells Proc Natl Acad Sci USA 2005 18 767 72 10.1073/pnas.0408932102 PMC545521 15640347

[B13] Deshmukh HS Case LM Wesselkamper SC Borchers MT Martin LD Shertzer HG Nadel JA Leikauf GD Metalloproteinases mediate mucin 5AC expression by epidermal growth factor receptor activation Am J Respir Crit Care Med 2005 15 305 14 10.1164/rccm.200408-1003OC 15531749

[B14] Shao MX Nakanaga T Nadel JA Cigarette smoke induces MUC5AC mucin overproduction via tumor necrosis factor-alpha-converting enzyme in human airway epithelial (NCI-H292) cells Am J Physiol Lung Cell Mol Physiol 2004 287 L420 L427 10.1152/ajplung.00019.2004 15121636

[B15] Boots AW Hristova M Kasahara DI Haenen GR Bast A van d V ATP-mediated activation of the NADPH oxidase DUOX1 mediates airway epithelial responses to bacterial stimuli J Biol Chem 2009 284 17858 67 10.1074/jbc.M809761200 19386603 PMC2719424

[B16] Richter A O'Donnell RA Powell RM Sanders MW Holgate ST Djukanovic R Davies DE Autocrine ligands for the epidermal growth factor receptor mediate interleukin-8 release from bronchial epithelial cells in response to cigarette smoke Am J Respir Cell Mol Biol 2002 27 85 90 12091250 10.1165/ajrcmb.27.1.4789

[B17] Williams TJ Jose PJ Neutrophils in chronic obstructive pulmonary disease Novartis Found Symp 2001 234 136 41 discussion 141-8 11199093 10.1002/0470868678.ch9

[B18] Stockley RA Neutrophils and the pathogenesis of COPD Chest 2002 121 5 Suppl 151S 5S 12010844 10.1378/chest.121.5_suppl.151s

[B19] De Boer WI Sont JK Van SA Stolk J Van Krieken JH Hiemstra PS Monocyte chemoattractant protein 1, interleukin 8, and chronic airways inflammation in COPD J Pathol 2000 190 619 26 10.1002/(SICI)1096-9896(200004)190:5<619::AID-PATH555>3.0.CO;2-6 10727989

[B20] Fuke S Betsuyaku T Nasuhara Y Morikawa T Katoh H Nishimura M Chemokines in bronchiolar epithelium in the development of chronic obstructive pulmonary disease Am J Respir Cell Mol Biol 2004 31 405 12 10.1165/rcmb.2004-0131OC 15220136

[B21] Pauwels RA Buist AS Calverley PM Jenkins CR Hurd SS Global strategy for the diagnosis, management, and prevention of chronic obstructive pulmonary disease. NHLBI/WHO Global Initiative for Chronic Obstructive Lung Disease (GOLD) Workshop summary Am J Respir Crit Care Med 2001 163 1256 76 11316667 10.1164/ajrccm.163.5.2101039

[B22] Investigative use of bronchoscopy, lavage and bronchial biopsies in asthma and other airways diseases J Investig Allergol Clin Immunol 1991 1 271 7 1688292

[B23] Lordan JL Bucchieri F Richter A Konstantinidis A Holloway JW Thornber M Puddicombe SM Buchanan D Wilson SJ Djukanovic R Holgate ST Davies DE Cooperative effects of Th2 cytokines and allergen on normal and asthmatic bronchial epithelial cells J Immunol 2002 169 407 14 12077271 10.4049/jimmunol.169.1.407

[B24] Slebos DJ Ryter SW van der TM Liu F Guo F Baty CJ Karlsson JM Watkins SC Kim HP Wang X Lee JS Postma DS Kauffman HF Choi AM Mitochondrial localization and function of heme oxygenase-1 in cigarette smoke-induced cell death Am J Respir Cell Mol Biol 2007 36 409 17 17079780 10.1165/rcmb.2006-0214OC PMC1899328

[B25] Borger P Vellenga E Gringhuis SI Timmerman JA Lummen C Postma DS Kauffman HF Prostaglandin E2 differentially modulates IL-5 gene expression in activated human T lymphocytes depending on the costimulatory signal J Allergy Clin Immunol 1998 101 2 Pt 1 231 40 9500757 10.1016/s0091-6749(98)70388-4

[B26] Heijink IH Kies PM Kauffman HF Postma DS van Oosterhout AJ Vellenga E Down-regulation of E-cadherin in human bronchial epithelial cells leads to epidermal growth factor receptor-dependent Th2 cell-promoting activity J Immunol 2007 178 7678 85 17548604 10.4049/jimmunol.178.12.7678

[B27] Kwon OJ Au BT Collins PD Adcock IM Mak JC Robbins RR Chung KF Barnes PJ Tumor necrosis factor-induced interleukin-8 expression in cultured human airway epithelial cells Am J Physiol 1994 267 4 Pt 1 L398 L405 7943343 10.1152/ajplung.1994.267.4.L398

[B28] Nakanaga T Nadel JA Ueki IF Koff JL Shao MX Regulation of interleukin-8 via an airway epithelial signaling cascade Am J Physiol Lung Cell Mol Physiol 2007 292 5 L1289 L1296 10.1152/ajplung.00356.2006 17220369

[B29] Sahin U Weskamp G Kelly K Zhou HM Higashiyama S Peschon J Hartmann D Saftig P Blobel CP Distinct roles for ADAM10 and ADAM17 in ectodomain shedding of six EGFR ligands J Cell Biol 2004 164 769 79 10.1083/jcb.200307137 14993236 PMC2172154

[B30] Marshall LJ Ramdin LS Brooks T DPhil PC Shute JK Plasminogen activator inhibitor-1 supports IL-8-mediated neutrophil transendothelial migration by inhibition of the constitutive shedding of endothelial IL-8/heparan sulfate/syndecan-1 complexes J Immunol 2003 171 2057 65 12902511 10.4049/jimmunol.171.4.2057

[B31] Endo K Takino T Miyamori H Kinsen H Yoshizaki T Furukawa M Sato H Cleavage of syndecan-1 by membrane type matrix metalloproteinase-1 stimulates cell migration J Biol Chem 2003 278 40764 70 10.1074/jbc.M306736200 12904296

[B32] Kudo T Takino T Miyamori H Thompson EW Sato H Substrate choice of membrane-type 1 matrix metalloproteinase is dictated by tissue inhibitor of metalloproteinase-2 levels Cancer Sci 2007 98 563 8 10.1111/j.1349-7006.2007.00426.x 17425593 PMC11159475

[B33] Kode A Yang SR Rahman I Differential effects of cigarette smoke on oxidative stress and proinflammatory cytokine release in primary human airway epithelial cells and in a variety of transformed alveolar epithelial cells Respir Res 2006 7 132 10.1186/1465-9921-7-132 17062156 PMC1634855

[B34] Hurtado M Lozano JJ Castellanos E Lopez-Fernandez LA Harshman K Martinez A Ortiz AR Thomson TM Paciucci R Activation of the epidermal growth factor signalling pathway by tissue plasminogen activator in pancreas cancer cells Gut 2007 56 1266 74 10.1136/gut.2006.097188 17452424 PMC1954978

[B35] Langlois S Gingras D Beliveau R Membrane type 1-matrix metalloproteinase (MT1-MMP) cooperates with sphingosine 1-phosphate to induce endothelial cell migration and morphogenic differentiation Blood 2004 103 3020 8 10.1182/blood-2003-08-2968 15070679

[B36] Schneider D Ganesan S Comstock AT Meldrum CA Mahidhara R Goldsmith AM Curtis JL Martinez FJ Hershenson MB Sajjan U Increased cytokine response of rhinovirus-infected airway epithelial cells in chronic obstructive pulmonary disease Am J Respir Crit Care Med 2010 182 332 40 10.1164/rccm.200911-1673OC 20395558 PMC2921598

[B37] Schulz C Kratzel K Wolf K Schroll S Kohler M Pfeifer M Activation of bronchial epithelial cells in smokers without airway obstruction and patients with COPD Chest 2004 125 1706 13 10.1378/chest.125.5.1706 15136380

[B38] Tomaki M Sugiura H Koarai A Komaki Y Akita T Matsumoto T Nakanishi A Ogawa H Hattori T Ichinose M Decreased expression of antioxidant enzymes and increased expression of chemokines in COPD lung Pulm Pharmacol Ther 2007 20 596 605 10.1016/j.pupt.2006.06.006 16919984

