# Supplementary figures and images for: Role of aberrant metalloproteinase activity in the pro-inflammatory phenotype of bronchial epithelium in COPD
Source: Respir Res. 2011 Aug 23;12(1):110. doi: 10.1186/1465-9921-12-110 (PMC3182910; doi:10.1186/1465-9921-12-110)

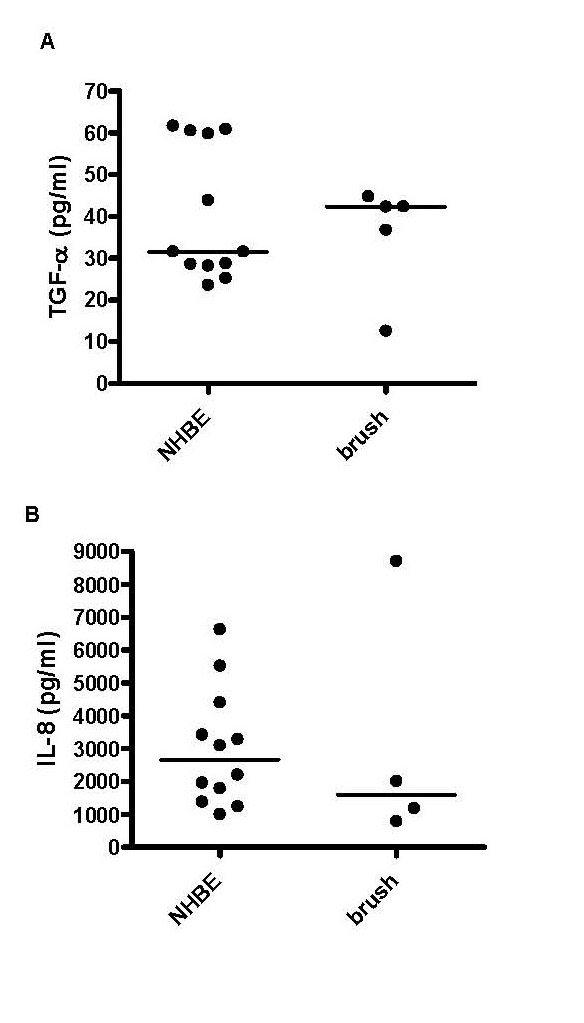

Supplement: Additional file 1 — Baseline TGF-α and IL-8 levels are not significantly different between epithelial cells (healthy smokers and non-smokers) obtained by bronchial brushings (brush) and normal human bronchial epithelium (NHBE) derived from Lonza. PBEC were growth factor-deprived overnight, pre-treated and incubated with medium. A) TGF-α levels at 2 hrs. Absolute values and medians are shown. B) IL-8 levels at 24 hrs. Absolute values and medians are shown. [file 1465-9921-12-110-S1.JPEG]

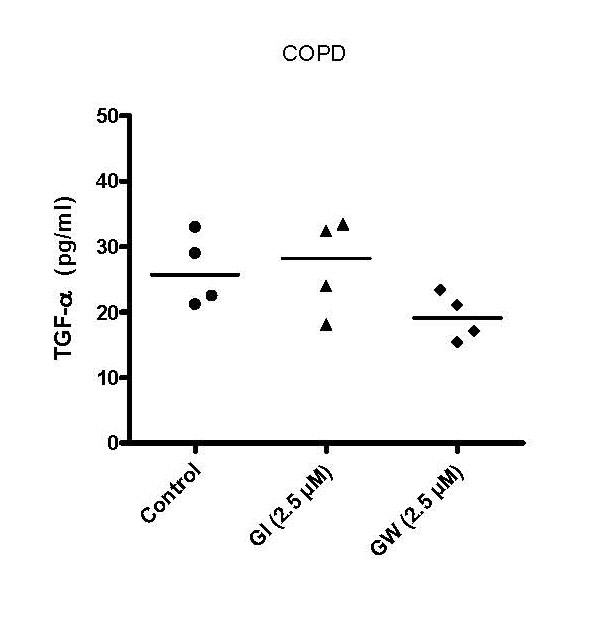

Supplement: Additional file 2 — TGF-α shedding in PBECs from COPD patients. PBECs were growth factor-deprived overnight and treated with and without GI254023 (2.5 μM) or GW280264 (2.5 μM) for 2 hrs. Absolute TGF-α levels and medians are shown. [file 1465-9921-12-110-S2.JPEG]

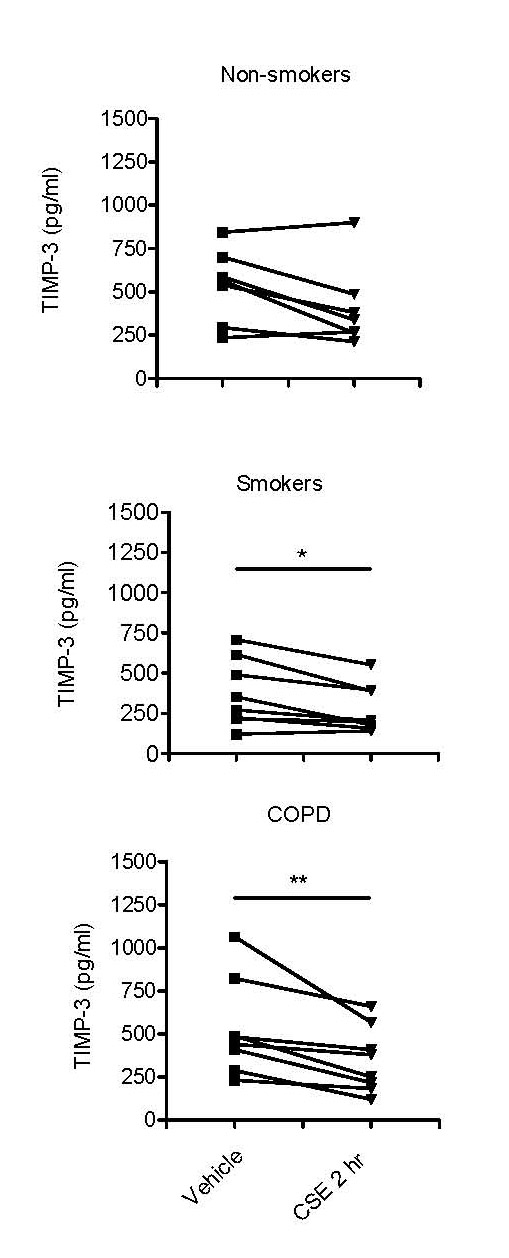

Supplement: Additional file 3 — TIMP-3 levels are significantly reduced by CSE exposure in PBECs from smokers and COPD, but not from healthy controls. PBECs were cultured and seeded in duplicates. Cultures were growth factor-deprived overnight and directly harvested for total cell lysates or incubated for 24 hrs with and without 5% CSE. Absolute values are shown. Significance is indicated (* = p < 0.05 and p**=<0.01). [file 1465-9921-12-110-S3.JPEG]

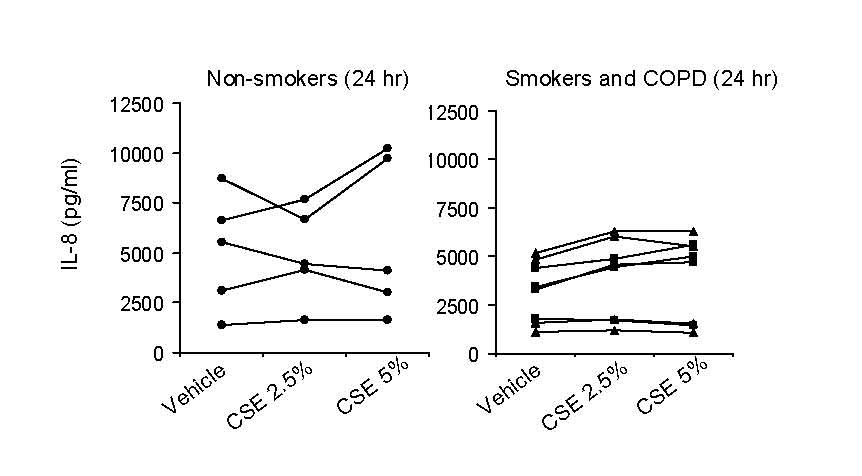

Supplement: Additional file 4 — IL-8 secretion in PBECs is not significantly different upon exposure to 5% CSE compared to 2.5% CSE. PBEC from non-smokers (circles), healthy smokers (squares) and COPD patients (triangles) were growth factor-deprived overnight and incubated with medium or CSE (2.5% and 5%) for 24 hrs. Exposure to 5% CSE does not further increase IL-8 levels in PBECs from smokers/COPD patients nor induce an increase in PBECs from non-smokers. Absolute values are shown. [file 1465-9921-12-110-S4.JPEG]

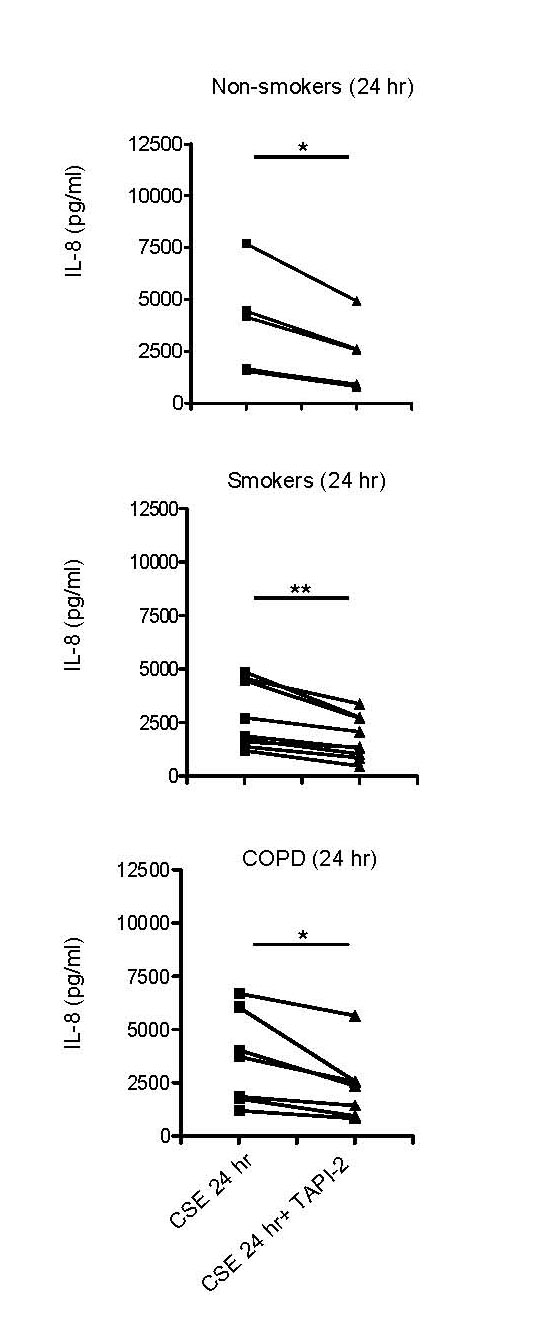

Supplement: Additional file 5 — CSE-induced IL-8 secretion in PBECs is significantly inhibited by TAPI-2. PBEC from non-smokers, healthy smokers and COPD patients were growth factor-deprived overnight, pre-treated with and without TAPI-2 (2.5 μM) and incubated with medium or 2.5% CSE for 24 hrs. TAPI-2 significantly inhibits CSE-induced IL-8 levels in PBEC. Absolute values are shown. Significance is indicated (* = p < 0.05 and ** = p < 0.01). [file 1465-9921-12-110-S5.JPEG]

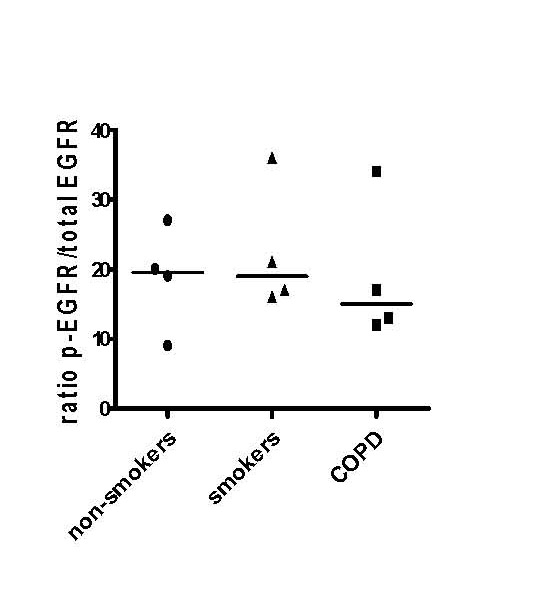

Supplement: Additional file 6 — Basal phospho-EGFR levels are not different between the subject groups. PBECs from non-smokers (n = 4), healthy smokers (n = 4) and COPD patients (n = 4) were growth-factor deprived overnight and total cell lysates were prepared. EGFR and phospho-EGFR detected by western blotting using anti-phospho-EGFR (1173 tyrosine residue) and anti-EGFR (Santa Cruz Biotechnology, Santa Cruz, CA). Densitometry was performed and (phospho-)EGFR levels were related to EGFR. Ratios and medians are depicted. [file 1465-9921-12-110-S6.JPEG]

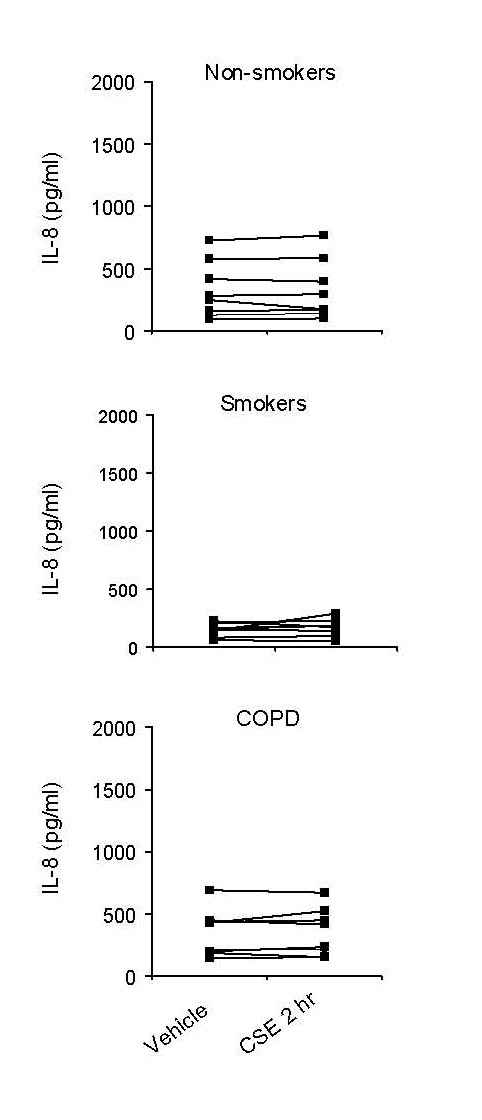

Supplement: Additional file 7 — Early levels of IL-8 are not induced by CSE in PBECs. Cells were growth factor-deprived overnight and incubated with medium or 2.5% CSE for 2 hrs. CSE has no effect on IL-8 levels in PBEC from the 3 different subject groups. Absolute values are shown. [file 1465-9921-12-110-S7.JPEG]
